# Effects of caffeine on reaction time are mediated by attentional rather than motor processes

**DOI:** 10.1007/s00213-017-4790-7

**Published:** 2017-12-23

**Authors:** Christopher W. N. Saville, H. M. de Morree, Neil M. Dundon, S. M. Marcora, C. Klein

**Affiliations:** 1 North Wales Clinical Psychology Programme, School of Psychology, Bangor University, Adeilad Brigantia, Ffordd Penrallt, Bangor, Gwynedd, Wales LL57 2AS UK; 2 Personal Health Department, Philips Research, Eindhoven, The Netherlands; 3 Department of Child and Adolescent Psychiatry, Psychotherapy, and Psychosomatics, Medical Faculty, University of Freiburg, Freiburg, Germany; 4 School of Sport and Exercise Science, University of Kent, England, UK; 5 Department of Child and Adolescent Psychiatry, Psychosomatics and Psychotherapy, Medical Faculty, University of Cologne, Cologne, Germany

**Keywords:** Caffeine, P300, Single trial analysis, Mediation, Event-related potentials, Reaction times

## Abstract

**Background:**

Caffeine has a well-established effect on reaction times (RTs) but the neurocognitive mechanisms underlying this are unclear.

**Methods:**

In the present study, 15 female participants performed an oddball task after ingesting caffeine or a placebo, and electroencephalographic data were obtained. Single-trial P3b latencies locked to the stimulus and to the response were extracted and mediation models were fitted to the data to test whether caffeine’s effect on RTs was mediated by its effect on either type of P3b latencies.

**Results:**

Stimulus-locked latencies showed clear evidence of mediation, with approximately a third of the effect of caffeine on RTs running through the processes measured by stimulus-locked latencies. Caffeine did not affect response-locked latencies, so could not mediate the effect.

**Discussion:**

These findings are consistent with caffeine’s effect on RTs being a result of its effect on perceptual-attentional processes, rather than motor processes. The study is the first to apply mediation analysis to single-trial P3b data and this technique holds promise for mental chronometric studies into the effects of psychopharmacological agents. The R code for performing the single trial analysis and mediation analysis are included as supplementary materials.

**Electronic supplementary material:**

The online version of this article (10.1007/s00213-017-4790-7) contains supplementary material, which is available to authorized users.

Caffeine has been the subject of great interest as a possible cognitive enhancer. One of its most consistently replicated cognitive effects is its reduction of reaction times (RTs) in speeded tasks (e.g. Childs and De Wit 2006; Haskell et al. 2005; Heatherley et al. 2005; Mclellan et al. 2016). What is less clear, however, is what neurocognitive mechanisms are behind this effect.

RTs measure the total duration of orienting to stimuli, identifying said stimulus, choosing the appropriate response, converting this response into a motor plan, and executing the motor plan. Caffeine’s influence on RTs could result from accelerating any, or all, of these stages.

On a neurochemical level, the psychomotor effects of caffeine have been shown to be mediated by its antagonistic binding to adenosine receptor sites (Snyder et al. 1981). Pharmacological studies in rats suggest that its effect on RTs may be due to its antagonistic effect on A_2A_ adenosine receptors specifically (Higgins et al. 2007). However, again in rats, adenosine receptor antagonists have been shown to both stimulate motor behaviour (Karcz-Kubicha et al. 2003) and to reduce attentional lapses (Christie et al. 2008), suggesting that caffeine could affect either the attentional or motor sub-processes of RTs, or indeed both. There is also evidence from human studies that caffeine’s effect on RTs may be driven by broad noradrenergic effects on alertness (Smith et al. 2003), as well as specific effects on stimulus encoding (Smith et al. 1999), that do not appear to be adrenergic (Smith et al. 2003)**.**

On a neurocognitive level, event-related potentials (ERPs) are an ideal technique to identify which of the sub-processes underpinning RTs are affected by caffeine. The P3b, a positive deflection over midline parietal cortex that is prominent in response to ‘oddball’ stimuli in choice RT tasks, is a promising candidate ERP component for this purpose. It has been suggested that the P3b acts as a bridge between stimulus-evaluation and response-planning sub-processes of the RT (Verleger et al. 2005). The P3b is unusual among ERP components in that it appears in both stimulus-locked and response-locked ERPs, consistent with this proposed bridging role. In terms of what this ‘bridging’ might represent in mechanistic terms, there in interesting evidence that the peak of the P3b represents the reaching of a perceptual decision threshold, distinct from motor-specific motor planning (O’Connell et al. 2012). Across these frameworks, we can ascribe a different significance to the stimulus and response-locked P3b latencies; the former appears to index the duration of perceptual/cognitive decision-making while the latter appears to reflect the time taken to convert such a decision into a motor plan and execute it.

A number of ERP studies have investigated caffeine effects on the P3b, but have largely focused on amplitude effects. Taking a mental chronometric approach (Kutas et al. 1977; Wagenmakers and van der Maas 2008), the effect of caffeine on P3b latency has more obvious theoretical implications than its effect on amplitude. Diukova et al. (2012) found that caffeine reduced stimulus-locked P3b latency, while Martin and Garfield (2006) found no effect of caffeine on stimulus-locked P3b latency. We are not aware of any studies examining the effect of caffeine on response-locked P3b latency.

To date, ERP studies of the effects of caffeine have used average ERPs. While this is a powerful way to improve signal-to-noise ratio of electroencephalographic data, it should not be forgotten that average ERPs treat all inter-trial variability as noise to be averaged out, regardless of whether this variability is meaningful or not. It is possible to measure the single-trial P3b (Saville et al. 2011) and single-trial P3b latencies predict RTs on corresponding trials (Philiastides and Sajda 2006), demonstrating that inter-trial variation is indeed meaningful. Indeed both stimulus and response-locked P3b latencies have been shown to predict RTs, in line with the idea that they represent the latencies of different processing stages (Saville et al. 2015a). Given that the single-trial P3b may thus be closer to the underlying phenomenon than the averaged P3b, it would be instructive to identify the possible effects of caffeine on the single-trial P3b.

Furthermore, in a statistical, and logical, sense, in order to confirm the neurocognitive locus of caffeine’s effect as the processes underlying the P3b, it would not be enough to show that (a) caffeine affects RTs and (b) caffeine affects P3b latency; caffeine’s effects on P3b latency and RT may be entirely separate. It must additionally be shown that the effect of caffeine on RT is *mediated* by P3b latency.

A mediating variable, *M*, is a third variable which lies between a predictor variable, *A*, and a dependent variable, *B*. The effect of *A* on *B* is thus not direct, but partly or entirely due to the effect of *A* on *M* and *M* on *B*. This type of relationship can be established by fitting a series of regression models to the data showing that *A* predicts *M* and *B*, *M*, predicts *B*, but the predictive power of *A* on *B* is partially or entirely abolished by controlling for *M*.

Single-trial P3b latencies and RTs are ideal data to use in a mediation analysis as mediation methods are now able to accommodate data nested within participants, allowing greatly increased statistical power to be brought to bear, relative to using only averaged ERPs and mean RTs.

The present study aims to test whether caffeine’s effect on RTs is mediated through stimulus and/or response-locked P3b latencies by fitting regression-based mediation models to both types of single-trial P3b latencies.

## Methods

All procedures were approved by the ethics committees of the School of Psychology and of the School of Sport, Health, and Exercise Science at Bangor University. Participants gave written informed consent prior to all testing.

### Participants

Eighteen female participants (Age 21.6 ± 4.1, one left-handed) took part in this study. Data from two participants were excluded for having fewer than 20 clean trials and those from a third were excluded due to a technical problem with data collection, leaving a final total of 15 participants’ data. All reported having no neurological or psychiatric diagnoses and were non-smokers. Four took oral contraceptives and one used a NuVaRing. Participants reported average caffeine use of 124 ± 109 mg/day.

### Apparatus

Fifty-nine Ag/AgCl ring electrodes in a 10–10 montage and two infra-orbital electrodes were used to record direct-current EEG. Prior to collecting data, impedance at each electrode was reduced to ≤ 5 kΩ using Abralyt high-chloride gel (EasyCap, Germany). Cz and FPz were used as the recording reference and ground electrodes respectively. Two BrainAmp DC amplifiers amplified the data before it was digitised and recorded using BrainVision Recorder (both Brain Products, Germany). Stimuli were presented on a 17″ LCD monitor with an electrically-shielded power source and the whole recording occurred in a sound-attenuated Faraday cage.

### Procedure

Data presented here were collected during the same sessions as a study into the effects of caffeine on the cortical correlates of perceived effort, during isometric leg contractions. For the sake of brevity, the full procedure of that study will not be reported here, except where relevant, but the interested reader is referred to de Morree et al. (2014) for further details.

Participants were recruited for two EEG sessions, exactly 1 week apart. The study employed a randomised counterbalanced crossover design whereby participants were administered caffeine during one session and placebo in the other. Caffeine was administered via capsules containing 6 mg/kg body weight of caffeine powder and 6 mg/kg body weight of dried milk, while in the placebo condition participants were given capsules containing 12 mg/kg body weight of dried milk. Both the participant and the experimenter were blind to condition. As participants might recognise the effects of caffeine, effectively unblinding them and reducing the potency of the placebo effect, they were told that they would be given caffeine in one session and taurine in the other. Participants were debriefed and told the truth after both sessions were complete.

Participants were asked to maintain their habitual levels of caffeine use throughout the testing period and to have a good night’s sleep before each session. They were also asked to avoid alcohol and intense exercise prior to each session, and to eat a light meal about 2 h prior to testing.

The capsules were administered prior to electrode attachment and the cognitive task described here was conducted after the leg contraction task. The mean duration between capsule administration and the start of the cognitive task was 2 h and 6 min (range 1:50–3:02).

The cognitive task used was a three-stimulus oddball design. Participants saw a series of coloured circles (~ 3 cm diameter) appear on a black background for 500 ms. The majority were white (standards 70%), but a minority were green (targets 15%) or red (distractors 15%). Participants were asked to make a left-handed keyboard response for all white circles, a right-handed keyboard response for green circles, and no response to red circles. Stimuli were presented in three blocks of 340 trials. The inter-trial interval varied uniformly between 1350, 1475 1600, 1725, and 1850 ms.

### Data analysis

A script for the portion of our analysis conducted in R (R Core Development Team 2012) is available as supplementary materials to this paper. Readers who would like any clarification on this script are very welcome to contact the corresponding author.

Each session’s data were preprocessed using BrainVision Analyser 2 (Brain Products, Germany). All three blocks were concatenated and sections where amplitude ranged by < .5 μV or > 1500 μV in any 200 ms window were excluded. Independent components analysis (ICA) using the Infomax algorithm was run on a 3-min stretch of data, starting a minute into each dataset, and weightings derived from the ICA were applied to the whole dataset. Components reflecting electro-ocular or electro-cardiographic artefacts were removed before data were back-projected. Channels showing significant residual artefacts limited to just that channel were interpolated using spherical splines (order = 3, Legendre polynomials = 10) before data were average referenced and .05–50.00 Hz filters (25 dB/octave roll-offs) were applied to the data. A second artefact rejection stage with more stringent criteria (amplitude ranging > 150 μV in any 200 ms window) was run to exclude any data still contaminated by artefacts.

An additional 4 Hz (25 dB/octave roll-offs) filter—shown to be optimal for single-trial analysis (Smulders et al. 1994)—was then applied to the data and data from target trials, correctly responded to between 120 and 1000 ms, were cut into segments from 600 ms pre-stimulus until 1800 ms post-stimulus. Data were baseline corrected using the period 600–400 ms pre-stimulus. Shorter stimulus (600 ms pre-stimulus to 1400 ms post-stimulus) and response-locked (700 ms pre-response to 400 ms post-response) segments that shared a common baseline were then cut from the longer segments. These data were then exported to R (R Core Development Team 2012) for single-trial analysis.^1^

**Fig. 1:**
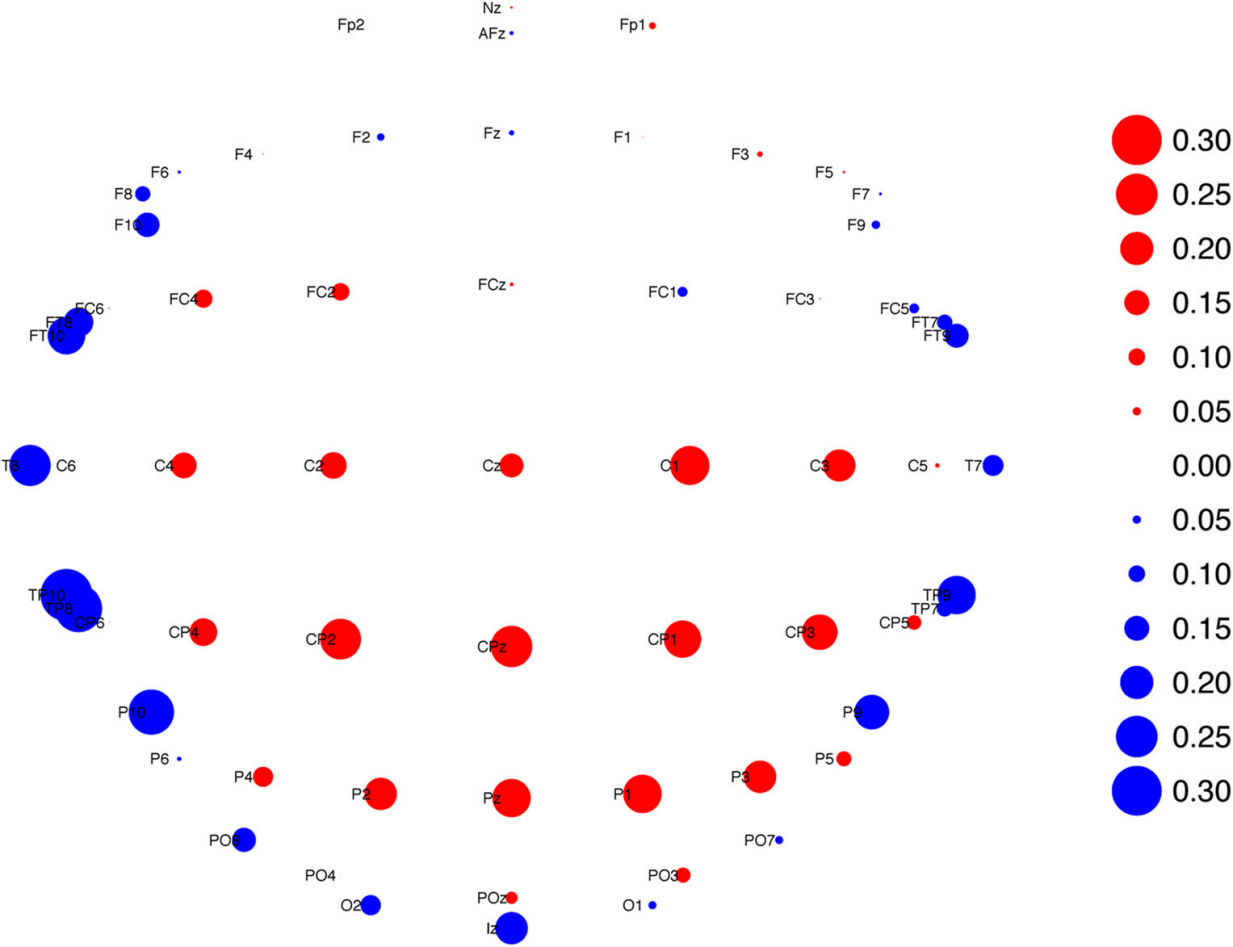
Topography of first infomax-rotated PCA factor used in subsequent analyses. Size of point reflects weighting of each electrode with positive weightings shown in red and negative in blue. Some locations modified slightly to prevent overlapping points

Single-trial analysis was run using the same approach reported in Saville et al. (Saville et al. 2011, 2012, 2015a), but repeated here. Averaged stimulus-locked ERPs for each participant on each condition were computed using the single-trial data and these averages were concatenated along the time axis. Spatial principal components analysis (Dien 2010a) with Infomax rotation, implemented using the prcomp and infomax functions from the core stats and GPA rotation (Bernaards and Jennrich 2005) R packages, respectively. Six factors were retained based on a parallel scree test, as recommended for principal components analysis of EEG data by Dien (2010b), implemented using the fa.parallel function from the psych package (Revelle 2016). Factor 1 showed a P3b topography so data from all electrodes were summed at each timepoint, weighted by Factor 1’s loadings, to produce single virtual electrode time-courses reflecting this factor’s activity. The topography of Factor 1 is displayed in Fig. 1 (this figure and all others, was made using the ggplot2 package for R (Wickham 2009)).

Peaks were identified for each trial as the time-point with maximal amplitude 250–750 ms post-stimulus for stimulus-locked data, and 250 ms pre-response to 250 ms post-response for response-locked data.^2^ Trials where (a) the stimulus or response-locked peak was identified at the very first or last millisecond of the peak picking window; or (b) response-locked peaks occurred before stimulus-onset; were excluded as they likely reflected an misidentified peak. Before models were fitted to data, P3b latencies were centred and scaled by z-scoring latencies within participant separately.

Mixed effects models, implemented using the lme4 (Bates et al. 2012) package for R (R Core Development Team 2012), were used to test that the assumptions for mediation were met, namely that caffeine predicted P3b latency and P3b latency predicted RT. For this purpose, a model predicting P3b latency with a fixed effect of condition (caffeine/placebo) and a random slope of condition for each participant^3^ was compared to a null model omitting the fixed effect but with the same random effects structure. Likewise a model predicting RT with a fixed effect of P3b latency and a random intercept and slope of P3b latency for each participant^4^ was compared to a null model with no fixed effects. Both comparisons were made using Aikake information criteria. Mediation assumptions were tested separately for stimulus and response-locked P3b latencies.

Mediation analysis was conducted using the *mediation* package (Tingley et al. 2014) for R. A mediation model was fitted to the data predicting RT on a single-trial basis using the predictor of condition (placebo = 0, caffeine = 1) and centred P3b latency as a mediating variable. The inputs to this model were two linear mixed effects models. The first predicted RT with fixed effects of condition and P3b latency, with random intercepts and a random slope of P3b latency for each participant^5^ (the model did not converge when a random slope for condition was added). The second predicted P3b latency using a fixed effect of condition, with a random intercept for each participant^6^ (again, models including a random slope for condition did not converge). Both models used maximum likelihood estimation. We planned to fit separate mixed effects models for stimulus and response-locked P3b latencies if the assumptions for mediation were met.

Model-based mediation analysis was used to estimate the average direct effect (ADE —the effect of caffeine on RT after controlling for P3b latency) and the average causal mediation effect (ACME – the total effect of caffeine on RT minus the direct effect). Quasi-Bayesian Monte Carlo simulation was used to derive 95% confidence intervals for these parameters (Imai et al. 2010).^7^

We also conducted two control analyses to assess possible confounds. Firstly, with analyses of single-trial latencies it is important to assess whether there are amplitude differences between conditions as this could lead to different signal-to-noise ratios for peak picking in the two conditions, complicating interpretations of apparent latency effects. To do this, a linear mixed effect model was fitted to P3b amplitudes with a main effect of caffeine and a random intercept and slope of caffeine for each participant. This model was compared to a null model omitting the fixed effect using Aikake information criteria.

Secondly, when determining peak picking windows for stimulus and response-locked peaks, there are three options:

1. One can define the two sets of windows separately for stimulus and response-locked analyses. This allows the identified P3b peak to differ for the two analyses, which can mean that for a given trial the stimulus and response-locked P3b latency do not sum to the RT. However, it ensures that the windows are consistent relative to their time-locking events and that the measurement of both latencies is independent of RT (see below).
2. One can use the same stimulus-locked window for both types of peak. This means that in trials with very fast RTs the window for response-locked peaks is much wider post-RT than pre-RT and in very slow trials the window is much wider pre-RT than post-RT. This confounds measurement error in RT and response-locked P3b latencies, meaning that using the latter to predict the former violates the assumption of independence for regression.
3. One can use the same response-locked window for both types of peak. This has the opposite effect of option 2—stimulus-locked windows are moved forward for fast RTs and backwards for slow RTs—again violating independence assumptions.

The safest option to address these issues is run all three analyses and check whether results hold across all three. In addition to our main analysis, which used independent windows, we computed inferred response-locked latencies by subtracting RTs from stimulus-locked latencies, and inferred stimulus-locked latencies by adding RTs and response-locked latencies. The mediation models were also fitted to these data in order to assess whether the same pattern held for inferred latencies. Again, these inferred latencies were centred prior to model fitting.

Finally, in order to see what value single trial analysis added, compared to traditional averaged ERPs, mediation models were fitted to peak latencies obtained from average ERPs of factor 1 computed from the same trials as the single trial analysis was conducted on. The mediation models were the same as used for single trial analysis only they used a single mean RT for each participant in the place of RTs for all trials and the peak latency picked from the average RT in the place of single trial peaks. A random intercept of participant was fitted (a random slope of condition would have yielded more parameters than data-points). Again separate analyses were run for stimulus and response-locked data.

## Results

**Fig. 2:**
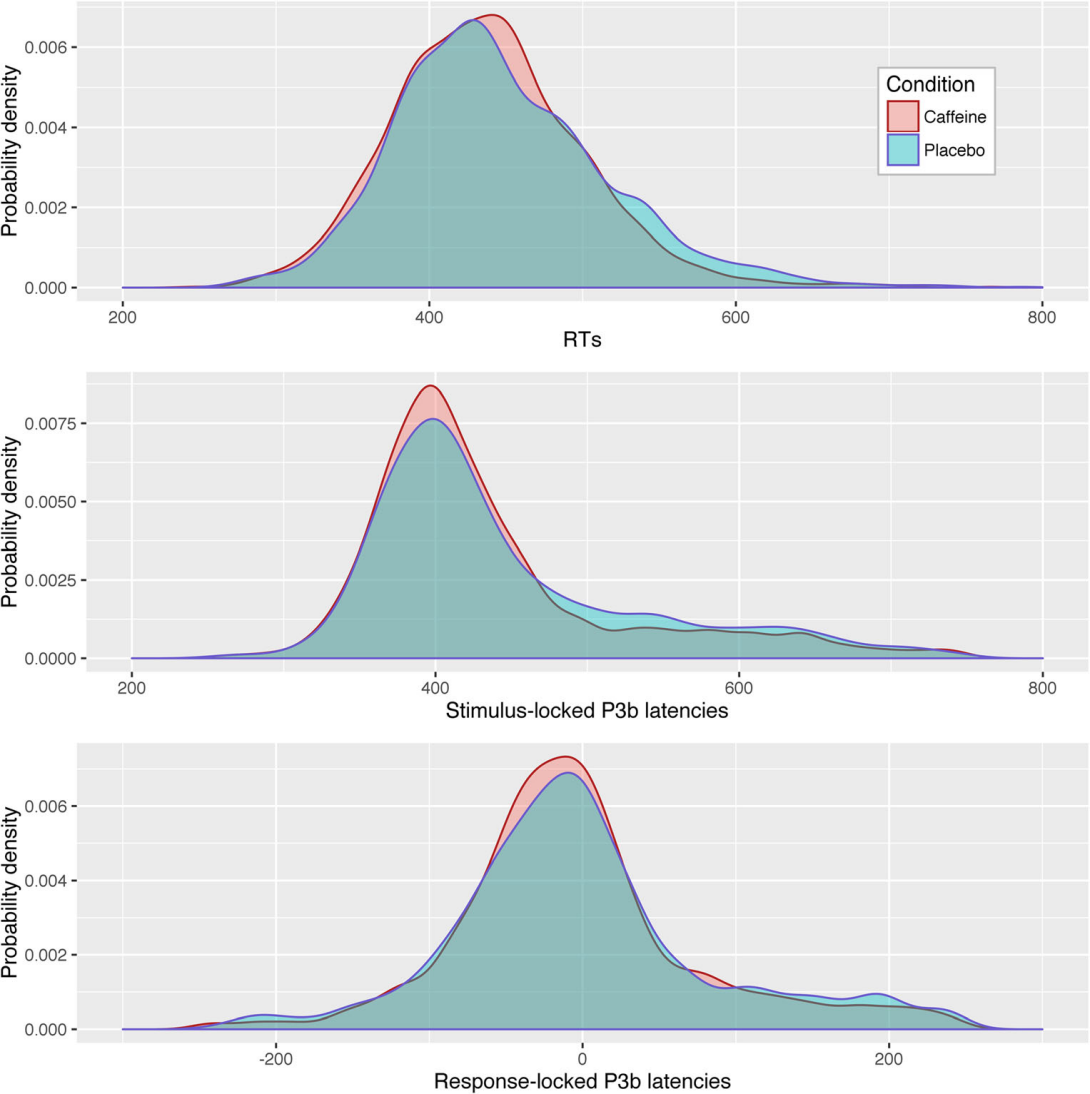
Probability density plots for RTs, stimulus-locked P3b latencies, and response-locked P3b latencies

Descriptive statistics for RTs; and stimulus and response-locked P3b latencies, and amplitudes can be found in Table 1. Probability density plots for the two sets of latencies and RTs can be found in Fig. 2.

**Table 1:** Descriptive statistics for RTs, and stimulus and response-locked P3b latencies and amplitudes. Variables for which models including Condition outperformed null models are indicated with asterisks in Condition effect row. Such models were fitted to single trial data, rather than means, as described in the manuscriptᅟ

	Mean RT	Mean stimulus-locked P3b latency	Mean response-locked P3b latency	Mean stimulus-locked P3b amplitude	Mean response-locked P3b amplitude
Caffeine	436.4 ± 33.4	439.6 ± 25.4	−2.806 ± 23.9	47.69 ± 16.6	47.67 ± 16.6
Placebo	448.2 ± 41.7	448.6 ± 33.7	2.459 ± 32.5	49.85 ± 17.0	49.89 ± 16.9
Condition effect	*	*

### Assumption checks

The model testing that condition predicted stimulus-locked P3b latencies (*B* = −.14, *B*_σ_ = .04, *t* = −3.54; *A* = .03, *A*_σ_ = .02, *t* = 1.31, *R* ^2^_m_ = .006, *R* ^2^_c_ = .011)^8^ was a better fit for the data than the null model omitting the fixed effect (AIC = 8269.898 vs 8278.166, *χ* ^2^ = 10.27, *p* = .001). The model testing that stimulus-locked P3b latencies predicted RTs (*B* = 25.90, *B*_σ_ = 2.42, *t* = 10.72; *A* = 442.76, *A*_σ_ = 8.76, *t* = 50.56, *R* ^2^_m_ = .122, *R* ^2^_c_ = .384) = was a better fit for the data than the null model omitting the fixed effect (AIC = 33,878.83 vs 33,908.60, *χ* ^2^ = 31.77, *p* < .001). Thus the assumptions for mediation were met for stimulus-locked P3b latency.

The model testing that condition predicted response-locked P3b latencies (*B* = −.04, *B*_σ_ = .04, *t* = −1.10; *A* > .01, *A*_σ_ = .02, *t* = −0.06, *R* ^2^_m_ < .001, *R* ^2^_c_ = .005) was not an improvement on the null model (AIC = 8308.016 vs 8307.217, *χ* ^2^ = 1.20, *p* = .273). The model testing that response-locked latencies predicted RTs (*B* = −57.48 *B*_σ_ = .97, *t* = −59.18; *A* = 409.94, *A*_σ_ = 8.69, *t* = 47.16, *R* ^2^_m_ = .280, *R* ^2^_c_ = .866) was, however a good fit for the data (AIC = 31,585.87 vs 34,183.08, *χ* ^2^ = 2599.2, *p* < .001). Thus the assumptions for mediation were *not* met for response-locked P3b latencies and this analysis was not run.

**Fig. 3:**
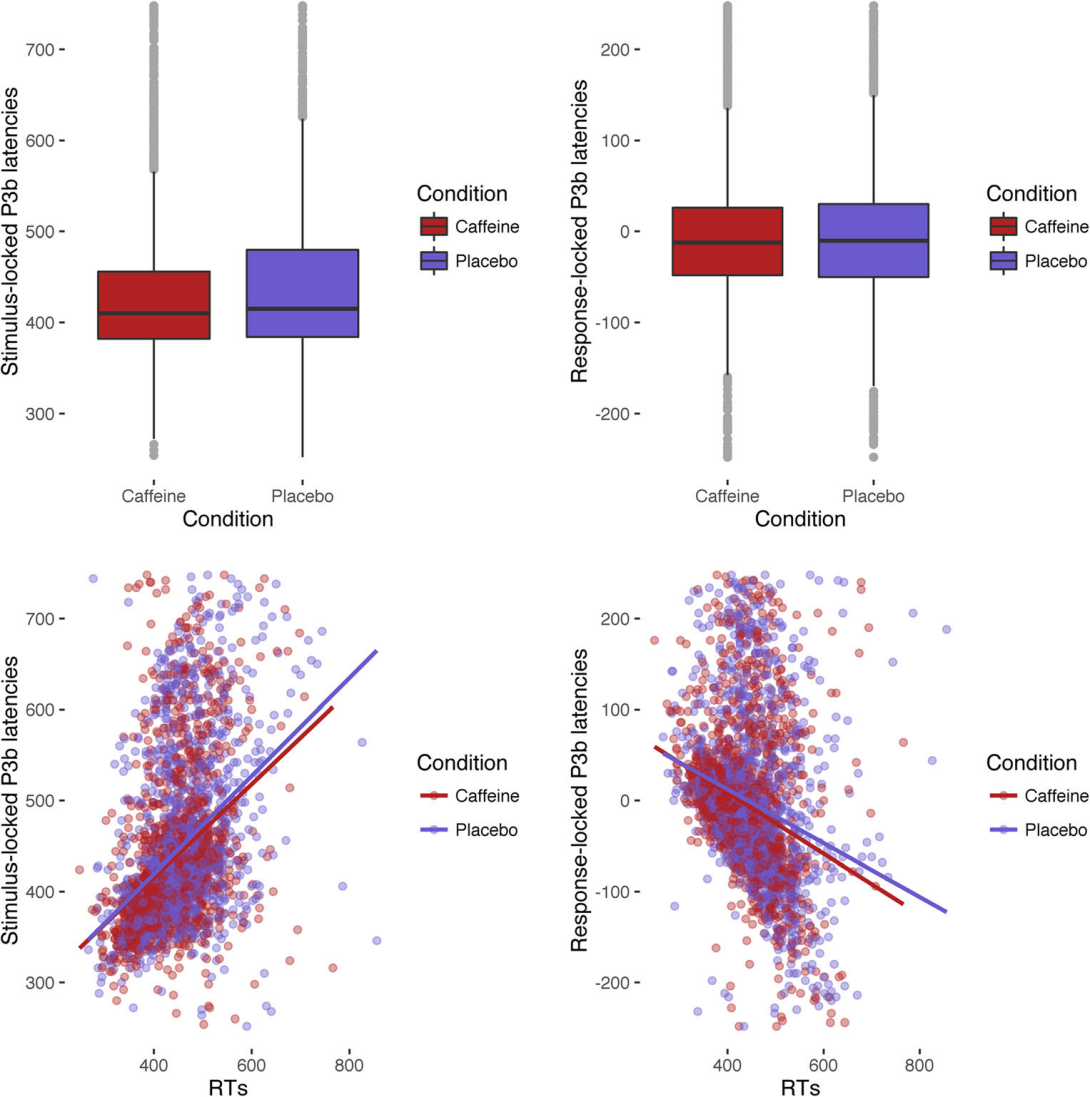
Assumption checks for mediation analysis. Boxplots compare single-trial P3b latencies, centred within participant, between conditions. Scatter plots show relationships between P3b latencies and RTs

Plots depicting these assumption checks can be seen in Fig. 3. Boxplots show shorter stimulus-locked latencies in the caffeine condition, but no difference between conditions for response-locked latencies. Strong relationships exist between both latency types and RTs.

### Mediation analysis

**Fig. 4:**
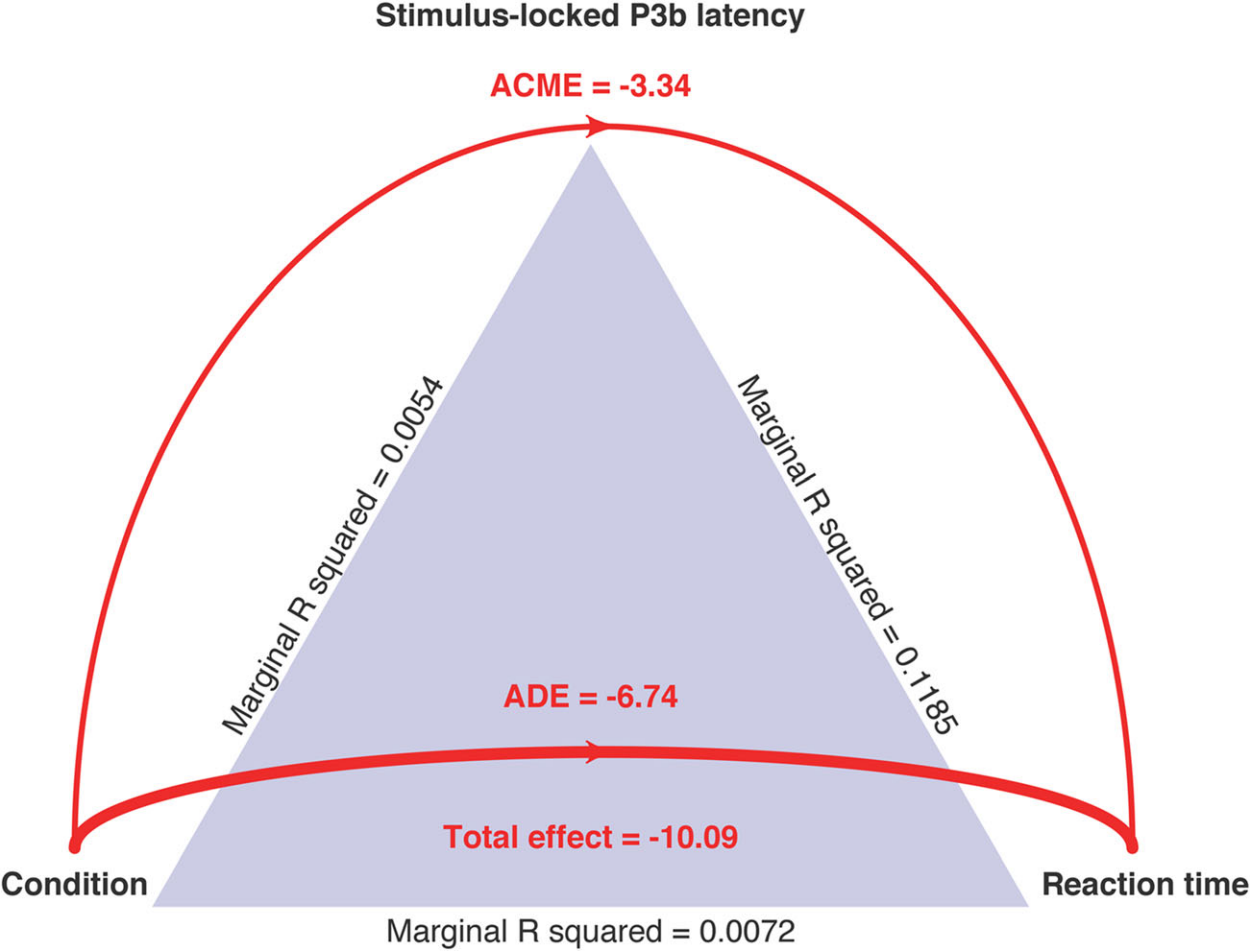
Mediation model for stimulus-locked P3b latency data. Marginal R2 values derived from mixed effect models fitted to test assumptions plus model predicting RT using condition with random intercept for each participant. Width of ACME and ADE lines weighted by proportion of effect mediated and unmediatedᅟ

The mediation model for stimulus-locked P3b latencies showed that the effect of caffeine was significantly mediated through its effect on single-trial P3b latencies. The ACME parameter was − 3.48 (CI = − 5.30–− 1.85, *p* < .01), the ADE was − 6.75 (CI = − 10.51–− 2.93, *p* < .01), the total effect of condition on RTs was − 10.23 (CI = − 14.34–− 6.06, *p* < .01), and the proportion of effect mediated was .34 (CI = .19–.57, *p* < .01). These results are presented in Fig. 4.

### Control analyses

The model predicting P3b amplitudes with condition was outperformed by the model with only the random effects (AIC = 28,806.87 vs 28,806.27, *χ* ^2^ = 1.40, *p* = .236), suggesting that caffeine did not meaningfully affect P3b amplitudes. This means that effects on latencies can be interpreted without concerns about differences in signal-to-noise ratio, due to caffeine effects on amplitudes. Amplitude data can be seen in Table 1.

Fitting mediation models to inferred latencies yielded a very similar result to the models fitted on directly measured latencies. The conditions for mediation were met for stimulus-locked latencies: condition predicted latencies (*B* = −.16, *B*_σ_ = .04, *t* = −4.03; *A* = .07, *A*_σ_ = .02, *t* = 2.97, *R* ^2^_m_ = .006, *R* ^2^_c_ = .09) and latencies predicted RTs (*B* = 40.99, *B*_σ_ = 1.99, *t* = 20.64; *A* = 452.42, *A*_σ_ = 8.75, *t* = 51.72, *R* ^2^_m_ = .122, *R* ^2^_c_ = .384). The effect of condition was significantly mediated through latencies: The ACME parameter was − 4.65 (CI = − 6.98–− 2.56, *p* < .01), the ADE was − 5.27 (CI = − 8.79–− 1.75, *p* < .01), the total effect of condition on RTs was − 9.91 (CI = − 14.16–− 5.76, *p* < .01), and the proportion of effect mediated was .47 (CI = .28–.74, *p* < .01). As with the directly measured response-locked peaks, their inferred equivalents did not meet the assumptions of mediation in that condition did not predict latency (*B* > − .01, *B*_σ_ = .04, *t* = − 0.08; *A* > .01, *A*_σ_ = .02, *t* = −.37, *R* ^2^_m_ < .001, *R* ^2^_c_ = .001). So, other than a slightly stronger mediation via stimulus-locked latencies than with the directly measured data, the results were essentially identical and the difference between results cannot be attributed to different peaks being identified in the two types of window.^9^

**Fig. 5:**
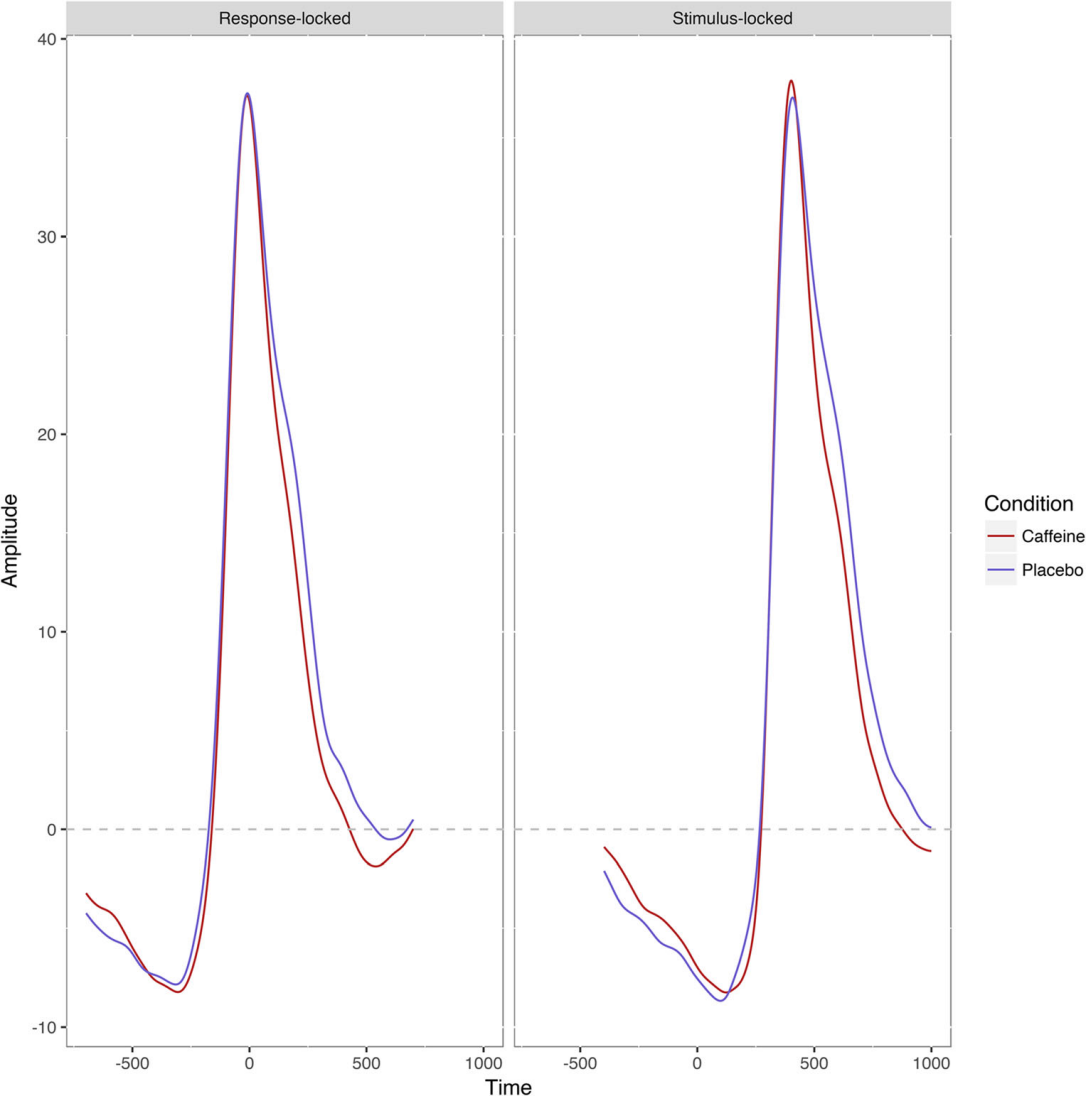
Average event-related potentials of stimulus and response-locked Factor 1 data for the two conditions. Note that amplitude is based on PCA-weighted sum of raw amplitudes, so units are arbitrary

Traditional grand averaged ERPs for the two conditions can be seen in Fig. 5. As with single-trial analyses, for stimulus-locked data, condition predicted latencies (*B* = −8.93, B_σ_ = 3.98, *t* = −2.24; *A* = 411.47, *A*_σ_ = 6.11, *t* = 67.36, *R* ^2^_m_ = .036, *R* ^2^_c_ = .795) and latencies predicted RTs (*B* = .66, *B*_σ_ = .26, *t* = 2.542; *A* = 174.56, *A*_σ_ = 107.27, *t* = 1.63, *R* ^2^_m_ = .160, *R* ^2^_c_ = .829). However, the mediation model was not significant (although it trended in that direction): ACME was −4.59 (CI = − 12.87–− .57, *p* = .098), the ADE was − 9.18 (CI = − 21.97–− 3.83, *p* = .16), the total effect of condition on RTs was − 13.77 (CI = −26.89–− .72, *p* = .039), and the proportion of effect mediated was .30 (CI = − .18–1.61, *p* = .13).

For response-locked data, neither assumption of mediation was met: condition did not predict latency (*B* = 4.93, *B*_σ_ = 12.42, *t* = 0.40; *A* = − 12.40, *A*_σ_ = 8.78, *t* = − 1.41, *R* ^2^_m_ < .001, *R* ^2^_c_ = .00) and latency did not predict RT (*B* = − .15, *B*_σ_ = .14, *t* = 1.07; *A* = − 442.93, *A*_σ_ = 9.24, *t* = 47.95, *R* ^2^_m_ = .002, *R* ^2^_c_ = .742).

## Discussion

We fitted mediation models to single-trial P3b latency and RT data, to test whether the effects of caffeine on RTs was mediated through stimulus or response-locked P3b latency. There was clear evidence of mediation for stimulus-locked latencies, with approximately a third of caffeine’s effect on RTs being due to its effects on stimulus-locked P3b latencies. Caffeine, however, did not affect response-locked P3b latencies and so cannot be a mediator for the effect on RTs. This effect was robust to inferring stimulus and response-locked P3b latencies from each other, suggesting that the different findings for the two latencies cannot be attributed to methodological artefacts from non-overlapping windows.

Caffeine did not affect P3b amplitude, in line with some studies of average ERPs (De Pauw et al. 2015; Tieges et al. 2004), but in contrast to others which show an increase in amplitude after caffeine consumption (Dixit et al. 2006; Martin and Garfield 2006; Ruijter et al. 2000). This is an interesting result in its own right, but in the context of single-trial analysis it is important because it suggests that differences between conditions in terms of latency cannot be attributed to differences in signal-to-noise ratio. Our single-trial analysis technique has identified effects on amplitude in some previous studies (Saville et al. 2011, 2014 ), but not others (Saville et al. 2015b, 2016) suggesting that it is sensitive to such effects when they are present.

Our findings suggest that processes that underlie stimulus-locked P3b latencies can be accelerated by caffeine and that a significant portion of caffeine’s effect on RTs can be attributed to this effect. In contrast, the processes underlying response-locked P3b latencies are not sensitive to caffeine. It is important to consider, however, that response-locked P3b latencies are at least as good predictors of RT as stimulus-locked latencies are, so the lack of an effect of caffeine is not because the processes underlying response-locked latencies are not important for RTs. In contrast, the increase in RT variability exhibited in attention-deficit hyperactivity disorder (ADHD) has been shown to be driven by greater variability in response-locked P3b latencies (Saville et al. 2015a), suggesting that the neurocognitive mechanisms underlying caffeine’s effect on RT are distinct from those associated with ADHD.

In terms of which neurocognitive mechanisms appear to be sensitive to caffeine, our findings are more consistent with effects on stimulus-processing, attentional lapses, and decision-making, rather than response-selection, motor planning, or motor execution. Caffeine has been shown to affect motor behaviour, via its antagonism of adenosine action, but the motor behaviour measured by Karcz-Kubicha et al. (2003) was the running of rats around their cage, which is not equivalent to the more motoric neurocognitive mechanisms we are concerned with here. As mentioned above, the present data were collected during the same session as study examining caffeine effects on neural correlates of perceived effort during a isometric knee extension task (de Morree et al. 2014) allowing us to draw direct comparisons with these results. In this study, caffeine had an effect on motor-related cortical potentials during movement execution, but again the task demanded a very different type of motor behaviour than the button presses required here. So while clearly caffeine can affect some types of motor behaviour, its effect on RTs does not appear to operate via this mechanism.

Our results are interesting for speculating about the processing model underlying RTs and the role of the processes underlying the P3b in this process. At first glance, our results seem consistent with some degree of independence of stimulus-locked and response-locked processing. Caffeine appears to aid cognitive processing as to speed up RT and stimulus-locked P3b peak latency without having an effect on response-locked P3b latency. A more parallel model of RT processing might expect an improvement of relatively early attentional processing to allow response-related motor preparation to begin earlier, speeding up response-locked P3b latencies also. However, it is important to remember that conceptualising the RT as being divided into pre-P3b and post-P3b stages is an oversimplification. As Fig. 2 shows, although a majority of response-locked P3b latencies occur prior to response, a substantial minority of peaks actually occur after responses. Whether or not this reflects simple error in peak identification, that scalp P3b peaks systematically lag the actual timing of important processing being completed, or whether it suggests that the processing underlying the P3b does not necessarily need to be complete prior to responding remains an interesting open question.

Although we find our results persuasive, and they were robust to the control analyses described above, the study had limitations. Firstly, we only used a single task and its demands were largely attentional. A task which was more demanding of motor-planning and execution might show an effect of caffeine on these sub-processes; indeed such a task did show an effect in the same session (de Morree et al. 2014). Against that, it is worth restating that response-locked P3b latencies were, if anything, a better predictor of RT than stimulus-locked latencies, which seems contrary to the notion that these processes were unimportant in this task. Secondly, we focus on the P3b and do not examine other components. This is defensible given the hypothesis-driven nature of our analysis, the depth of our analysis, and the risks of ‘fishing’ when examining too many dependent variables. However, our focus on the P3b may mean we missed potentially important effects elsewhere. Thirdly, although we ran the single-trial analysis with the PCA-Infomax denoised peak picking approach we have used elsewhere and a further analysis using electrode Pz instead of the Infomax factor, obtaining highly comparable results, it is currently unclear how similar the results of different single trial analysis algorithms are. For the current state of the art comparing different techniques, the reader is referred to Ouyang et al. (2017). Fourthly, previous authors have identified specific effects of caffeine on a minority of slow RTs (e.g. Smith et al. 2013). Our analyses only looked at the effects of caffeine on the overall speed of the RT distribution—it may be interesting to examine whether caffeine administration changes the shape of the distribution and how this comes about. Fifthly, the sample size was modest, especially given that three participants’ data were unusable. Power analysis in mediation is somewhat complicated, but it is likely that our study would not have been powered to detect mediation effects that were much subtler than the one observed.

From a methodological point of view, the present study is the first to apply mediation analysis to single-trial ERP data in this way, and this technique appears to have potential for the field of mental chronometry. The use of mixed effects models and single-trial analysis means that these models can be fitted to data from participant sample sizes that are feasible in psychopharmacological studies, so we believe the technique is an especially good fit for this field. Interestingly, similar models fitted to average ERPs did not detect a mediation effect, although the term trended towards significance, suggesting that our technique enjoys superior statistical power to existing methods.

To conclude, our mediation analysis of single-trial P3b latencies suggests that caffeine’s effect on RTs is driven by its acceleration of attentional, as opposed to motoric, sub-processes. This technique appears to be a promising means for studying the neurocognitive effects of psychopharmacological agents in future studies.

## Electronic supplementary material

ESM 1 (R 14 kb)

ESM 2 (RTF 15 kb)

ESM 3 (PDF 36 kb)

## Footnotes

1 In subsequent footnotes, we give code for specific model fits in R. A complete script of our R analyses is available in the supplementary materials.

2 This is a shorter time window than our previous papers (e.g. Saville et al. 2016). However, given the relatively simple task, we compared mixed effects models predicting RT using peaks obtained using these windows and the longer windows (250–1000 ms, −375–375 ms). The model using the shorter windows had a higher marginal *R* ^2^ (.31) than the model using the longer windows (.16).

3 R code for this model:

4 R code for this model:

5 R code for this model:

6 R code for this model:

7 R code for mediation model:

8 A = intercept, B = coefficient. Marginal *R* ^2^ (*R* ^2^_m_), reflecting the variance explained by the fixed effects and the conditional *R* ^2^ (*R* ^2^_c_), reflecting variance explained by the whole model, were computed based on Johnson’s (2014) extension of Nakagawa and Schielzeth’s (2013) method.

9 In order to check that our results were not contingent on details of our processing pathway, we reran our original analysis with peaks picked from electrode Pz, rather than the PCA-derived factor. Otherwise processing was unchanged. The results were strikingly similar. The ACME parameter was − 3.35 (CI = − 5.09–− 1.70, *p* < .01), the ADE was − 6.74 (CI = − 10.53–− 2.96, *p* < .01), the total effect of condition on RTs was − 10.08 (CI = − 14.21–− 5.97, *p* < .01), and the proportion of effect mediated was .33 (CI = .18–.55, *p* < .01). We also reran the analysis without centring and rescaling each participant’s P3b latencies. Again, results were almost identical: The ACME parameter was − 3.70 (CI = − 5.81–− 1.83, *p* < .01), the ADE was − 6.53 (CI = − 10.21–− 2.83, *p* < .01), the total effect of condition on RTs was − 10.23 (CI = − 14.44–− 6.14, *p* < .01), and the proportion of effect mediated was .36 (CI = .20–.59, *p* < .01).
